# A Brave New World: Virtual Reality and Augmented Reality in Systems Biology

**DOI:** 10.3389/fbinf.2022.873478

**Published:** 2022-04-06

**Authors:** Berk Turhan, Zeynep H. Gümüş

**Affiliations:** 1 Department of Genetics and Genomic Sciences, Icahn School of Medicine at Mount Sinai, New York, NY, United States; 2 Faculty of Natural Sciences and Engineering, Sabanci University, Istanbul, Turkey; 3 Precision Immunology Institute, Icahn School of Medicine at Mount Sinai, New York, NY, United States

**Keywords:** virtual reality, augmented reality, visualization design, 3D, immersive 3D, multi-omics visualization, CAVE, systems biology

## Abstract

How we interact with computer graphics has not changed significantly from viewing 2D text and images on a flatscreen since their invention. Yet, recent advances in computing technology, internetworked devices and gaming are driving the design and development of new ideas in other modes of human-computer interfaces (HCIs). Virtual Reality (VR) technology uses computers and HCIs to create the feeling of immersion in a three-dimensional (3D) environment that contains interactive objects with a sense of *spatial presence*, where objects have a spatial location relative to, and independent of the users. While this *virtual* environment does not necessarily match the *real* world, by creating the illusion of reality, it helps users leverage the full range of human sensory capabilities. Similarly, Augmented Reality (AR), superimposes virtual images to the real world. Because humans learn the physical world through a gradual sensory familiarization, these immersive visualizations enable gaining familiarity with biological systems not realizable in the physical world (e.g., allosteric regulatory networks within a protein or biomolecular pathways inside a cell). As VR/AR interfaces are anticipated to be explosive in consumer markets, systems biologists will be more immersed into their world. Here we introduce a brief history of VR/AR, their current roles in systems biology, and advantages and disadvantages in augmenting user abilities. We next argue that in systems biology, VR/AR technologies will be most useful in visually exploring and communicating data; performing virtual experiments; and education/teaching. Finally, we discuss our perspective on future directions for VR/AR in systems biology.

## BACKGROUND

We see the world in three dimensions (3D), because we have binocular vision, meaning our left and right eyes see slightly different views of an object-a topic explored since Euclid’s 3^rd^ century BC *Optics* and physician Galen’s 2^nd^ century AD *On the Use of the Different Parts of the Human Body*, to da Vinci’s (1452–1519) *Trattato della Pittura (Art of Painting)*. The first device that produced 3D-effects utilized binocularity by using two mirrors centered at 45° reflecting specific images to each eye. Invented in the 1830s by Charles Wheatstone, it was called *the reflecting mirror stereoscope*, from the Greek *skopion* and *stereo (see solid)*. Conceptually, Virtual Reality (VR) was first described by computing pioneer Sutherland (Sutherland, 1965), who created arguably the first VR head-mounted display (HMD)—a large and bulky device that required mounting to the ceiling and could cause severe injury if it fell on a user, which was nicknamed the *Sword of Damocles* (Sutherland, 1968). However, the actual term *Virtual Reality* was coined later in the 1980s, when Visual Programming Lab of computer scientist Jaron Lanier began producing the first commercially available VR headsets and gloves. Since then, efforts to integrate the human body naturally into the virtual experience have driven significant advances in VR and Augmented Reality (AR) human-computer interfaces (HCIs), which mainly consist of input devices, output displays, and various hardware and software parts. We summarize these technologies in Table 1.

Notable within these efforts, CAVE Automatic Virtual Environments (CAVEs) were introduced in the 90s (Cruz-Neira et al., 1992) by projecting stereoscopic images on walls and floor of a room-sized cube. A CAVE can be thought of like Star Trek’s Holodeck (Chu and Quek, 2013), though the name refers to the metaphor of Plato’s cave in the *Republic* (Plato and Shorey, 1930) where a philosopher contemplates perception, reality, and illusion. Inside a CAVE, users wear Liquid-Crystal Display (LCD) shutter glasses and a head-tracker and interact with objects using a wand-like device to gain immersion that utilizes a full range of human vision with much wider field-of-view (FOV) and enhanced *perceptive depth* and *shape perception*. Later on, for massive data, large, ultra-high-resolution matrices of multiple displays (either monitors or projectors) called Powerwalls were also developed (Papadopoulos et al., 2015). Powerwalls and CAVEs are attractive as collaborative environments in which several investigators can simultaneously interact with VR objects. Yet, they are expensive to build, maintain and upgrade, occupy considerable space, and require many displays and massive processing power, making them cost-prohibitive. Thus, with these available technologies VR was not commercially popular to go mainstream (Wohlgenannt et al., 2020).

Until recently, VR was even considered a “dead technology” (Slater and Sanchez-Vives, 2016). However, recent technological advances, especially in gaming products including Oculus Rift (Menlo Park, CA), HTC VIVE (Taiwan), and PlayStation VR (San Mateo, CA), have enabled VR to finally have good enough performance at relatively cheaper prices, creating a positive feedback loop between companies that develop more advanced technologies and expanding consumer demand, which led to a tremendous jump in VR technology (Wohlgenannt et al., 2020; Kugler, 2021). For context, commercial VR systems in 2016 required expensive and difficult setups including an HMD headset, controllers, and sensors connected to an external high-graphics computer. However, some current new generation VR sets such as Oculus Quest 2 (Menlo Park, CA) and HTC VIVE Focus series (Taiwan), generally referred as *stand-alone VR systems*, or *all-in-one solutions*, do not even depend on any external computer system (Wohlgenannt et al., 2020), increasing accessibility further. Coupled with very recent developments such as the popular metaverse concept that combines multiple elements of VR/AR and internet technologies to achieve an extended reality by blending physical and digital worlds together (Lee et al., 2021), we anticipate that VR/AR technologies will finally go mainstream.

## VR/AR IN SYSTEMS BIOLOGY

In systems biology, we often seek to provide new insights that weave data on molecules, pathways, cells and tissues to whole organisms, populations, and ecosystems in multiple time- and length-scales. Furthermore, new high-throughput-omics experimental techniques are producing massive and diverse multi-omics datasets (Gillette et al., 2020; Kalayci et al., 2020; Petralia et al., 2020; Marx, 2021; Satpathy et al., 2021), and detailed data capture is boosted further by advances in supercomputers and tracking sensor technologies. Parallel increases in processor speeds and data storage enable computational analyses of these data (Schadt et al., 2010). New or updated visualization technologies are needed to explore and communicate these datasets. While not new in scientific visualizations (Bryson, 1996; Simpson et al., 2000), VR technologies offer novel avenues to these unprecedented new data communication needs. However, much remains to be answered on when and how to use VR/AR technologies in systems biology.

To help answer these questions for different use cases, we first provide a summary of the advantages and disadvantages of using VR technologies in Table 2. Notably, their greatest advantage is in providing *unparalleled presence*—the sense of being inside of and interacting with the virtual environment (Slater and Wilbur, 1997; Schubert et al., 2001; Sutcliffe et al., 2005). Therefore, we recommend utilizing VR/AR technologies when *spatial presence* and immersive interactivity with the content makes a difference in addressing user needs. User comprehension may become somewhat limited in 2D above domain-specific data type and size thresholds, leading to data occlusions. In such cases, immersion enables 3D-navigation and provides the necessary perceptive depth to enhance comprehension. For example, for multi-dimensional data, user studies have reported significantly better performance for immersive 3D vs. 2D environments for certain analysis tasks (Etemadpour et al., 2013). As researchers working at the interface of biology and data visualization, based on our own experience in this domain, and observations on the general trends in VR applications in biology that we briefly summarize here, we anticipate their utilization in three main areas in systems biology: 1) visually exploring and communicating data; 2) performing virtual experiments; and 3) education/teaching and discuss them below.

### Visually Exploring and Communicating Data

Systems biology visualizations are generally abstract representations, far from real-world objects. For instance, cellular pathway graphs are often cartoon representations. Yet, they suffice in helping understand the biological phenomena they represent. In VR environments, users interpret such abstractions as real objects, and the imagery has a lasting impact on our brains. Furthermore, we can interact with the virtual objects in ways that are not possible in the real world at multiple scales, from single molecules (Leinen et al., 2015), protein-drug complexes (Norrby et al., 2015) biomolecular networks (Liluashvili et al., 2017) to organs (Mirhosseini et al., 2014); navigate through them (Bellmund et al., 2016), or dynamics (Nakano et al., 2016). VR technologies thus open the door to benefit diverse phenomena that involve 3D spatial reconstructions, from localizations and dynamics in the human brain (Calì et al., 2016) to interactions in retinal pathology (Aaker et al., 2011) and volumetric studies in digital pathology (Farahani et al., 2016; Liimatainen et al., 2021). More recently, single-molecule localization microscopy (SMLM) in immersive VR has been used to visualize biological structures as point clouds at the molecular scale (e.g., vLUME by Spark et al., 2020). Some of these recent SMLM tools can be further extended to other similarly multidimensional spatially localized point clouds (e.g., Genuage tool by Blanc et al., 2020).

In systems biology, the knowledge, visualization, and exploration of related 3D structural data often play an important role. To explore and understand the function of macromolecules (proteins, RNA, and DNA) and their complexes from their 3D structural models, molecular graphics is shifting to VR (Tse et al., 2011; Nakano et al., 2016; Ratamero et al., 2018; Cassidy et al., 2020; Todd and Emsley, 2021). Some of these applications further integrate relevant structural knowledge with domain-specific (Norrby et al., 2015), or genomics datasets (Zhang et al., 2019). While some molecular viewers work in CAVE environments (Block et al., 2009), others utilize latest HMDs (Leinen et al., 2015), game engines such as Unity and gesture devices such as Kinect and Leap Motion (Probst and Reymond, 2018; Zhang et al., 2019) or even voice recognition (Sabir et al., 2013) to activate commands; or provide web-based VR without head-tracking or advanced interactions (Li et al., 2014; Cassidy et al., 2020). Parallel efforts are also on-going to study the chemical fingerprints of DrugBank compounds in VR environments (Probst and Reymond, 2018).

VR is useful even when exploring abstract systems-level data that do not contain 3D-localizations. For example, researchers often employ complex network visualizations which may require many viewpoints due to clutter, and navigation issues. Several studies suggest stereoscopy alone (Ware and Mitchell, 2008; Greffard et al., 2014; Kwon et al., 2015; Kwon et al., 2016) or combined with rotation (Sollenberger and Milgram, 1993) or motion cues (Ware and Mitchell, 2008) enhances performance in comprehension, helps present graphs better than 2D-displays (Sollenberger and Milgram, 1993) and enables low user error rates (Ware and Mitchell, 2008). For example, Supplementary Figure S1 shows a relatively large network in 2D, and Supplementary Video S1 in 3D. While both are generated using the network visualization tool iCAVE (Liluashvili et al., 2017; Kalayci and Gümüş, 2018), within iCAVE users can further interactively explore the 3D or stereo versions from multiple perspectives. Visualizations from multiple perspectives reportedly make different aspects of a system more salient (Ellis et al., 1991). Similarly, the BigTop tool (Westreich et al., 2020) renders Manhattan plots from genome-wide association studies (GWAS) in 3D.

More recently, VR is used to explore multidimensional -omics datasets in systems biology including cytometry, transcriptomics, epigenomics, proteomics and their combinations, in the form of abstract data clouds. For example, single-cell RNA sequencing data analysis often includes a dimensionality reduction step, where cell populations are projected in 2D or 3D space to explore cellular heterogeneity. Visualizing such datasets in 3D can be more informative as it decreases the possibility of collapsing similar cell types and clusters. A recent tool, CellexalVR, allows visual exploration and analysis of such dimensionality reduction plots and associated metadata in immersive VR (Legetth et al., 2021). Other tools for the same purpose include starmapVR (Yang et al., 2020), singlecellVR (Stein et al., 2021) and Theia (Bressan et al., 2021). These platforms often include additional modalities such as on-the-fly clustering, or visualization of dynamic changes in RNA velocity. While singlecellVR and starmapVR are web applications that enable visualizations using low-cost and easily available VR hardware such as Google Cardboard (Yang et al., 2020; Stein et al., 2021), CellexalVR involves GPU-accelerated performance and in-session on-the-fly calculations. StarmapVR further enables simultaneous visualization of spatial transcriptomics data from matching histological images. We anticipate that in the near future we will witness further developments in VR tools for the visual exploration of spatial transcriptomics datasets.

### Performing Virtual Experiments

For research studies that cannot be performed in the real world, VR provides a safe, standardized, and reproducible environment that is as life-like as possible (Tarr and Warren, 2002). In addition, we can break the laws of optics and physics, or disconnect real life sensed by the user’s body from the world the user is experiencing (Tarr and Warren, 2002). Researchers have been using such VR properties to study, modify or enhance behavior. For example, neural processes research that links biology and behavior in different species, from insects to humans (Ravassard et al., 2013; Aghajan et al., 2015; Acharya et al., 2016) has used VR to help understand sensory cues that carry information on the virtual worlds or refine the rules that link a subject’s actions to changes in their world. In addition to assisting in understanding human behavior, research can in turn inform how VR environments can be improved in design for increased human engagement. For example, studies suggest that the socially networked nature of VR should be considered in tool design, as numbers of remote users in virtual spaces increase (Kruzan and Won, 2019; Jeong et al., 2021).

In systems biology, virtual experiments can help develop and improve scientific thinking skills. Virtual reformulations of experiments in the form of games to be solved have already proven efficiency in tackling scientific problems. For example, the tools Foldit (Foldit, 2022) and Eterna (Eterna, 2022) have gamified the protein folding and RNA structure prediction problems (Das et al., 2019), and thereby enabled more individuals to perform virtual experiments by engaging the online gaming community whose members may have little to no scientific knowledge. Yet, these gamers have successfully solved real-world problems in relatively short time scales (Cooper et al., 2010; Khatib et al., 2011; Eiben et al., 2012; Horowitz et al., 2016; Koepnick et al., 2019). Similarly, the tool EyeWire has gamified mapping neural circuits in the brain to understand vision, where players try to virtually map 3D neuron structures to serial electron microscopy image data from animal brains (Das et al., 2019; EyeWire, 2022). This game has so far attracted more than 150 K gamers who helped reveal 6 new neuron types and many undiscovered brain circuits (EyeWire Into the Brain, 2022). VR environments open exciting new possibilities of such gamification of virtual scientific problems both for scientists with little coding experience, as well as for non-scientists, and the communication between these two communities. We have already started to observe the first examples of such tools in computational chemistry, where Shannon *et al* have introduced molecular dynamics VR game to encourage users to explore the reactivity of a specific chemical system (Shannon et al., 2021), and an intuitive VR platform called Nanome presented by Kingsley et al., where the users explore and modify chemical structures collaboratively to work on structural biology and molecular drug design problems (Kingsley et al., 2019). The next several years will witness an increasing number of tools that gamify virtual experiments in the VR environments. Note that in online gaming communities, VR is increasingly blended with social media functionalities, and thus gamified systems biology VR tools will likely need to consider such additional functionalities that are critical for remote users.

### Education/Teaching

VR environments can help learning in systems biology areas that involve complex 3D information (Salzman et al., 1999; Mikropoulos and Natsis, 2011), user-interactivity and/or high computational skills. For example, understanding protein structure traditionally involves physical modeling kits or projections of the 3D structures into 2D. However, the ability to create, alter, and rotate a chemical structure in real time in 3D can make it easier to understand abstract concepts (Limniou et al., 2008). Similarly, annotated 3D web-based anatomy atlases help teach complex structures such as artery networks or bronchial trees. Earlier interactive 3D-renderings of these systems used desktops with standard screens due to the costs of VR processors and displays (Li et al., 2012), while later technologies have enabled stereoscopic immersive 3D with real-time interactivity (Kockro et al., 2007). Randomized user studies show that stereo-enhanced 3D-tools are useful in learning anatomy and are well-received by students (Kockro et al., 2015; de Faria et al., 2016). Similarly, integrating AR technology reportedly has positive impact on student learning in biology (Weng et al., 2020).

Recent technological advances in VR have substantially increased their potential utility in learning. Biological concepts currently constitute ~5% of academic publications on VR (Morimoto and Ponton, 2021). Relatively popular educational platforms include those that simulate biological and chemical experiments within VR environments, such as VRLab Academy (United Kingdom), Labster (Denmark), and ClassVR (United Kingdom). Advances in gaming have expanded VR applications in education as well. For example, the tool Peppy provides a Unity-based VR gaming engine to understand protein structures and their dynamics in undergraduate biochemistry classes (Doak et al., 2020). Similarly, Pepblock Builder VR tool provides a gamified interface for researchers who are not advanced in the computational skills required for protein design (Yallapragada et al., 2021). Many educational VR experiences exist in popular gaming platforms that recreate biological systems, such as The Body VR, where players move in the bloodstream to observe human cells and learn how organelles function (The Body VR LLC, 2016), and InCell VR, where players fight to stop a virus invasion in human, while learning about cell and organelle microstructures (Luden.io, 2015). We anticipate that gaming-based VR tools will similarly be developed for learning multi-omics datasets at a systems level. Further research will then be necessary to understand the full potential impact of VR in learning systems biology.

## DISCUSSION

The recent explosion in VR/AR technologies has coincided with extended work-from-home practices due to the coronavirus disease 2019 global pandemic. These developments have lowered resistance to virtual technologies and in fact created a pressing need for virtual, collaborative workspaces in research. Coupled with the explosive increase in datasets collected from multi-omics high-throughput experiments, VR/AR technologies offer attractive new opportunities for visual data exploration and communication in systems biology. However, to develop the most useful tools, systems biologists need to conduct their own user studies and get familiar with design practices within virtual spaces for improved human perception (Cleveland and McGill, 1987). With deeper understandings of the brain and visual perception, content creation and best practices will be established, and adoption will increase. Further technological improvements (higher frame rates, efficient information storage and rendering; increased data transfer with less bit rates; game engines; graphics cards) will aid challenging visualizations such as dynamic networks or multi-scale systems, integrated with data annotations and on-the-fly calculations. Visualization outcomes will in turn guide future research protocols.

VR application development is already easier with HMDs and input devices that offer game engine-compatible free software development kits. These render information from internetworked devices that collect and exchange data with sensors and network connections (Rose, 2014; Akyildiz et al., 2015; Open Hybrid, 2022) or from integrating multiple technologies (e.g., healthcare in cyberspace (Rosen et al., 2016)). We anticipate that new technologies will further eliminate discomforts and limitations of modern VR headsets such as their bulkiness and weight. Towards this end, current studies include using skin sensory interfaces, such as nanowire-based soft wearable HMIs (Wang et al., 2021), and thin and light-weight holographic optics with high performance sunglasses-like near-eye full-color displays, as developed by Facebook Reality Labs (Maimone and Wang, 2020). Such new technologies may remove the barriers between the virtual and real worlds further by eliminating headset use, thereby converging VR/AR (Kugler, 2021).

In summary, we are currently at a critical juncture for VR/AR use in systems biology, as they are finally on the verge of going mainstream. We anticipate that the current trends towards utilizing low-cost VR/AR systems will continue. Still, for certain research areas, interactive 3D applications in Web3D will likely suffice. For some applications, AR will be preferable, as it allows users to overlap virtual data onto the real world in relatively simpler set-ups such as smartphones, without the need of HMDs, and provide more control of their surroundings (Garcia-Bonete et al., 2019). VR will likely remain advantageous in applications that require better immersion and realism (Garcia-Bonete et al., 2019). Barriers for access to VR/AR visualizations in systems biology will likely remain, however, for researchers from underdeveloped countries, and which will need to be addressed. At the same time, with the increasing trends in gamification within the VR environments (Shannon et al., 2021), barriers for scientists with low computational expertise and non-scientists in conducting their own virtual experiments will decrease. As user community grows and commercial VR/AR technologies expand, we expect the range of their systems biology applications will also continue to grow, opening possibilities for significant advancements in understanding and communicating disease-associated mechanisms, running virtual experiments, and education, and help boost the development of new therapies. Of course, the best way to gauge possibilities is to explore them!

## Supplementary Material

Supplementary Figure 1

Supplementary Material.docx

Supplementary Video 1

## Figures and Tables

**TABLE 1 |:** Input tracking and output display technologies in consumer-level VR/AR systems.

INPUT TRACKERS
Track user position (x, y, z) coordinates and orientation (yaw, pitch, roll angles) as users move about (either all or some)
“User position” may be body movements, head-rotations, or gestures
Feed tracking information back to the computer for real-time display updates (e.g., 3D mouse (wand) or data gloves)

**Types**
Generally use 3D computer vision, sync pulses and laser lines, or inertial measurement units to recognize movements, gestures and positioning to achieve intuitive tracking
**Full-body Tracking**
e.g., Microsoft Azure Kinect DK (Redmond, WA), Sony PlayStation Camera (San Mateo, CA), OptiTrack (Corvallis, OR), Intel RealSense Depth Cameras (Santa Clara, CA), OpenCV OAK (Palo Alto, CA), HTC Vive Tracker (Taiwan)
**Hand & Arm Motion Tracking**
e.g., Sony PlayStation Move (San Mateo, CA), Sony Dualsense Controller (San Mateo, CA), UltraLeap Trackers (United Kingdom)
**Eye and Facial Tracking**
e.g., Tobii Face Trackers (Sweden), VIVE Facial Tracker (Taiwan)

**Important Features**
*Update rate* (times/second) user position and orientation estimates sent to the computer
*Latency* user position and orientation recording and transmission time for display response
*Accuracy and Resolution* of user position and orientation
*Range* volume within which user position and orientation can be measured
*Ease of use, size, and weight*

OUTPUT DISPLAYS

Display a three-dimensional virtual world (usually stereoscopic) from the user’s eye positions, to create the perception that the virtual scene is independent of user movements

**Types**
**HMD systems**
*Higher Resolution (pixel density), high upgrade rate and low latency head-tracking*
*Render only user views instead of the entire scene, by placing small high-resolution displays directly in front of the user’s eyes, creating immersion at a fraction of the cost of larger displays*
e.g., HTC Vive (Taiwan); Microsoft Hololens (Redmond, WA); Oculus Rift and Quest (Menlo Park, CA); Sony Playstation VR (San Mateo, CA); Valve Index (Bellevue, WA); HP Reverb G2 (Palo Alto, CA)
**Commodity 3D TVs/stereo-enabled computers with low-cost 3D glasses and software**
*Low-cost*
*Limited to screen area*
*Human eye can utilize a much larger field-of-view (FOV)*
**Spatial Displays**
e.g., Sony Spatial Reality Display (San Diego, CA), Acer ConceptD 7 SpatialLabs (Taiwan)
**Mobile Devices**
e.g., iOS devices with ARKit (Cupertino, CA), Android devices with ARCore (Mountain View, CA)

HARDWARE AND SOFTWARE

Manage input/output; Analyze incoming data; Compute and render 3D-graphics based on input tracker feedback

**TABLE 2 |:** Advantages and Disadvantages of VR technologies.

ADVANTAGES
**1** *Unparalleled presence*—sense of being inside the virtual environment Slater and Wilbur, (1997); Schubert et al. (2001); Sutcliffe et al. (2005). Increased by:
**a** User movements and interactions Balakrishnan and Sundar, (2011), which enhance learning Cummings and Bailenson, (2016) and working memory-based performance McKendrick et al. (2016)
**b** User tracking, spectroscopy and wider FOV (significant increase) Cummings and Bailenson, (2016)
**c** Overall immersion (moderate increase) Cummings and Bailenson, (2016)
**d** Image quality and resolution (low increase) Lee, (2004)
**e** Update rate (potentially effective but is less studied)
**2** *Stereoscopic depth cues* are beneficial in exploring complex 3D/multidimensional 3D+ data Slater et al. (1996); Greffard et al. (2014); McIntire and Liggett, (2014). 2D-vis cannot provide spatial depth cues McIntire and Liggett, (2014)
*Increased Peripheral vision* provides a rich set of cues, permitting more natural and thus quicker interactive explorations of high dimensional and dynamic data
**3** *Full user attention*, as users cannot *second-screen* VR Wirth et al. (2007)
**4** *Complete stimuli control within a safe, standardized, and reproducible environment*, offering unprecedented opportunities for studies that cannot be performed in the real world Tarr and Warren, (2002)
**5** *Moving beyond keyboard/mouse*, supports natural modes for navigation or gestures such as flying/grasping
**6** Users can investigate multiple regions or explore correlations between data that are nearby in space-time without clutter, updated in real-time based on their movements and views
**7** *Collaborative environment*. Colleagues at multiple locations can meet, make eye contact (with their avatars), and explore objects together. As big data often involves many researchers in remote locations, this can help tremendously in team efforts

**DISADVANTAGES**

**1** HCI learning curves can pose barriers for new/occasional users (e.g., HMDs limit ability to interact with mouse/keyboard)
**2** User isolation from surroundings can cause a lack of situation awareness (imagine wearing an HMD on the subway). While see-through headsets can solve such problems, they are awkward to wear in public
**3** Some users may feel motion sickness/nausea, though increasing *peripheral vision* greatly reduces these
**4** Users may experience neck pain and stress if wearing for long periods
**5** Some technical & conceptual challenges need to be addressed to improve presence:
**a** Increased distance perception Renner et al. (2013)
**b** Quick updates with low latency
**c** Real-time delivery with high performance
**d** Rapid feedback on user actions at specific locations
**6** VR is unnecessary for systems that are simple, small, where additional depth cues may not be needed, and vital or useful

## Data Availability

The original contributions presented in the study are included in the article/Supplementary Material, further inquiries can be directed to the corresponding author.
