# Supplementary figures and images for: A Brave New World: Virtual Reality and Augmented Reality in Systems Biology
Source: Front Bioinform. 2022 Apr 6;2:873478. doi: 10.3389/fbinf.2022.873478 (PMC9140045; doi:10.3389/fbinf.2022.873478)

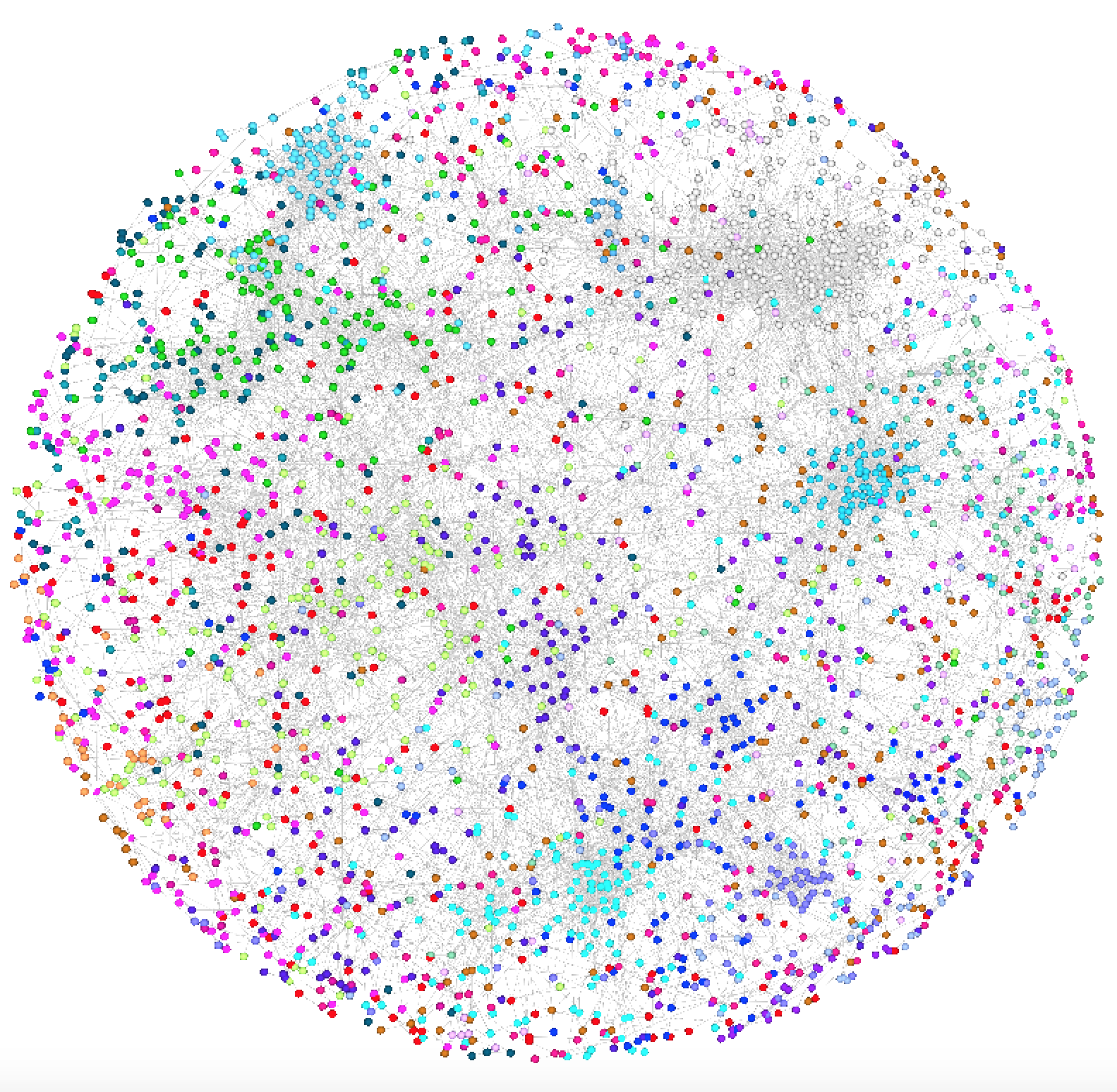

Supplement: Supplementary file 1 [file Image1.TIFF]
