# Supplementary material for: A Brave New World: Virtual Reality and Augmented Reality in Systems Biology
Source: Front Bioinform. 2022 Apr 6;2:873478. doi: 10.3389/fbinf.2022.873478 (PMC9140045; doi:10.3389/fbinf.2022.873478)
Supplement: Supplementary file 2 [file Table1.docx]

Supplementary Material

# Supplementary Data

**Supplementary Video 1.** Yeast genetic interaction network animation in 3D space. A yeast genetic interaction network animation generated by iCAVE (Liluashvili et al., 2017; Kalayci and Gümüş, 2018) using weighted force-directed layout algorithm in 3D space. Rotations of the visualization in 3D space makes it easier to inspect large numbers of interactions (and may even reveal interactions that are hidden in 2D space). A topological clustering is applied on the network and only those nodes belonging to clusters that exhibit discernible groupings (excluding the clusters that have proportionally large numbers of nodes) which are colored and scaled larger.

# Supplementary Figures and Tables

## Supplementary Figures


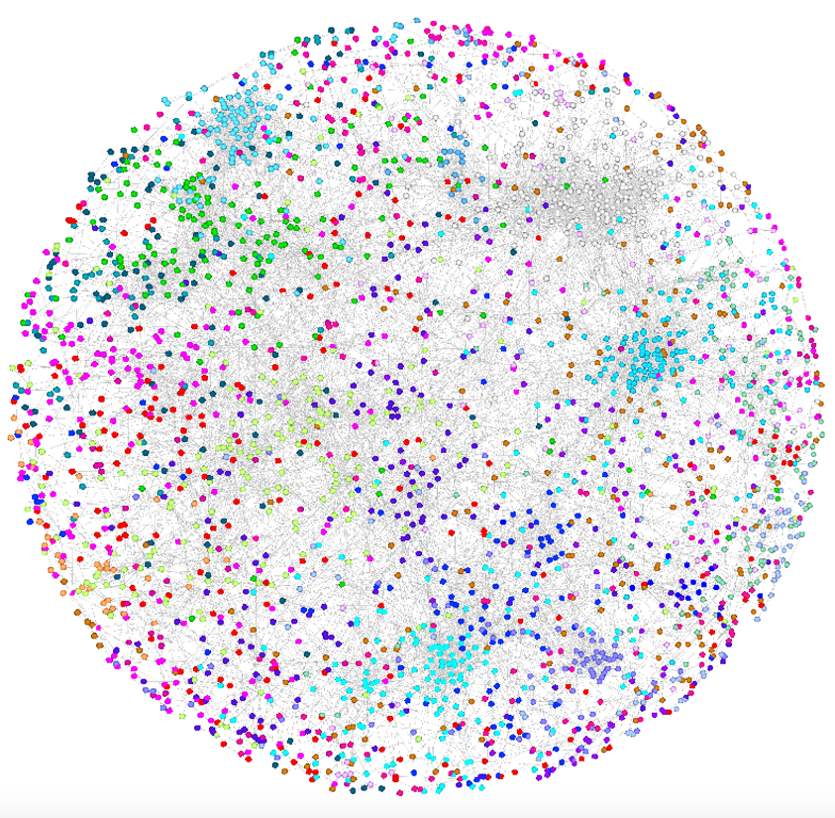


**Supplementary Figure 1.** An interaction network in 2D space. A 2D projection of a yeast genetic interaction similarity network visualized with iCAVE (Liluashvili et al., 2017; Kalayci and Gümüş, 2018). The network contains 2,838 nodes and 10,016 edges and has the same 2D coordinates from the original reference (Costanzo et al., 2010). Node colors represent topological clusters from the original reference.2D projection of the network flat across the display panel. Clearly, users cannot rotate/navigate within a 2D layout vs. the interactive 3D representation (Supplementary Video 1).

# References

Costanzo, M., Baryshnikova, A., Bellay, J., Kim, Y., Spear, E. D., Sevier, C. S., et al. (2010). The Genetic Landscape of a Cell. *Science* 327, 425–431. doi:10.1126/science.1180823.

Kalayci, S., and Gümüş, Z. H. (2018). Exploring Biological Networks in 3D, Stereoscopic 3D, and Immersive 3D with iCAVE. *Current protocols in bioinformatics* 61, 8.27.1-8.27.26. doi:10.1002/cpbi.47.

Liluashvili, V., Kalayci, S., Fluder, E., Wilson, M., Gabow, A., and Gümüş, Z. H. (2017). iCAVE: an open source tool for visualizing biomolecular networks in 3D, stereoscopic 3D and immersive 3D. *GigaScience* 6. doi:10.1093/gigascience/gix054.
